# Evaluation of Intraocular Anti-vascular Endothelial Growth Factor (VEGF) Drug-Associated Adverse Events Using Spontaneous Reporting Databases

**DOI:** 10.7759/cureus.91376

**Published:** 2025-09-01

**Authors:** Mugita Sato, Koichi Kageyama, Seiichiro Ito, Yoko Ino, Waranee Bunchuailua, Suang Rungpragayphan, Tomofumi Yamazaki, Kana Sugishita, Sineenart Krichanchai, Satoshi Nakao, Pornsak Sriamornsak, Hirofumi Takeuchi, Mitsuhiro Nakamura

**Affiliations:** 1 Laboratory of Drug Informatics, Gifu Pharmaceutical University, Gifu, JPN; 2 Laboratory of Pharmaceutical Health Care and Promotion, Gifu Pharmaceutical University, Gifu, JPN; 3 Health Consumer Protection and Pharmacy Administration, Silpakorn University, Nakohon Pathom, THA; 4 Biomedicine and Health Informatics, Silpakorn University, Nakohon Pathom, THA; 5 Faculty of Pharmacy, Silpakorn University, Nakohon Pathom, THA; 6 Laboratory of Advanced Pharmaceutical Process Engineering, Gifu Pharmaceutical University, Gifu, JPN

**Keywords:** aflibercept, age-related macular degeneration, japanese adverse drug event report database, ranibizumab, time-to-onset analysis

## Abstract

Introduction: Anti-vascular endothelial growth factor (VEGF) agents are the first-line therapies for macular edema, diabetic macular edema, and exudative age-related macular degeneration secondary to retinal vein occlusion. Although adverse events, including cerebral infarction, are rare, their potential severity warrants further investigation. This study aimed to evaluate the risk and time to onset of adverse events, including cerebral infarction, associated with anti-VEGF agents using the Japanese Adverse Drug Event Report (JADER) database.

Methods: Adverse events were coded using the Medical Dictionary for Regulatory Activities, and reporting odds ratios were calculated. The number of reports and median time to onset (interquartile range) were determined. Survival analyses were performed employing the log-rank test and the generalized Wilcoxon test.

Results: From April 2004 to January 2025, 965,286 reports were included in the JADER database. The number of adverse event reports for aflibercept (IVA), ranibizumab (IVR), brolucizumab (IVB), and faricimab (IVF) was 1,217, 1,223, 1,064, and 199, respectively. Furthermore, the reported numbers of cerebral infarction cases associated with IVA, IVR, IVB, and IVF were 200, 154, 16, and 20, respectively. The median (interquartile range) time to onset of cerebral infarction associated with IVA and IVR was 79.0 (range: 28.0-274.0) days and 48.5 (range: 14.0-142.5) days, respectively, with a significant difference in the time trend (log-rank test: p = 0.0100 and generalized Wilcoxon test: p = 0.0067).

Conclusion: The findings suggest that cerebral infarction may develop earlier with IVR use than with IVA use and highlight the need for careful monitoring and individualized treatment strategies in clinical practice.

## Introduction

Retinal vein occlusion and associated macular edema, diabetic macular edema, and exudative age-related macular degeneration (AMD) are significant ocular disorders associated with a risk of blindness. The number of patients with early and late AMD in the United States in 2019 was estimated to be 18.34 million and 1.49 million [1], respectively, and macular degeneration accounted for 9.1% of the causes of blindness in the Japanese population in 2019 [2]. AMD significantly impairs visual function, resulting in limited mobility and social participation, which may lead to feelings of social isolation. These psychosocial consequences can contribute to the development of mental health disorders such as depression, thereby reducing the overall quality of life. Moreover, the loss of visual independence poses considerable challenges in daily living and adversely affects the ability of elderly individuals to maintain autonomy [3]. Therefore, AMD constitutes a major issue not only in the medical domain but also from a broader social standpoint.

Vascular endothelial growth factor (VEGF) functions as a mitogenic factor for endothelial cells, promoting their migration, survival, and lumen formation, which are key processes involved in angiogenesis and vascularization [4-6]. Recently, intraocular administration of anti-VEGF agents has been found to be efficacious in the treatment of retinal vein occlusion, diabetic macular edema, and AMD. In Japan, four anti-VEGF agents are currently approved for intravitreal injection: aflibercept (IVA), ranibizumab (IVR), brolucizumab (IVB), and faricimab (IVF) [7]. As stated in the package inserts, all four anti-VEGF agents have been reported to be absorbed into the systemic circulation. Moreover, IVA, IVB, and IVF have been shown to decrease VEGF levels, while IVR has demonstrated only a modest tendency toward reduction [8-10]. Rare cases of embolic side effects of these agents [11] in individuals with AMD have been reported, and cerebral infarction is a significant risk factor in the affected patients [12]. Early detection of cerebral infarction is important because it becomes more severe with the passage of time [13,14]. These findings may play an important role in the risk assessment of and drug selection for cerebral infarction, a serious adverse drug reaction. However, few studies have compared the time of onset of cerebral infarction due to frequently used anti-VEGF agents.

Spontaneous adverse event reporting data, derived from real-world clinical practice and employed by regulatory authorities for pharmacovigilance purposes, are well suited for the analysis of rare adverse events, such as cerebral infarction associated with anti-VEGF agents. The Japanese Adverse Drug Event Report (JADER) database, maintained by the Pharmaceuticals and Medical Devices Agency (PMDA), contains spontaneous adverse event reports collected from clinical settings across Japan, including detailed information on the time of adverse event onset. Although numerous well-controlled clinical studies exist, understanding the occurrence of adverse events in the context of complex patient backgrounds and drug regimens in clinical practice remains essential. Analyses of adverse events associated with anti-VEGF agents using spontaneous reporting system databases are limited. To the best of our knowledge, this is the first study to investigate the association between anti-VEGF agents and cerebral infarction through outcome-based and time-to-onset analyses. This study aimed to evaluate the risk and time to onset of adverse events related to anti-VEGF agents using the JADER database. Furthermore, we specifically examined the occurrence of cerebral infarction associated with IVA and IVR.

## Materials and methods

Ethical approval

All results were obtained from publicly available data on the PMDA website [15]. The JADER database consists of fully anonymized reports, with all personal identifiers removed by regulatory authorities before data release. An ethical review was not required because this study was based on a secondary analysis of an existing public database and did not involve direct interactions with human subjects.

Data source

Adverse event reports in the JADER database were downloaded from the PMDA website. The database consists of four data tables: patient demographic information, such as sex and age (DEMO); drug information, such as generic name, purpose of administration, dose administered, association with adverse events, and the start and end dates of administration (DRUG); information on adverse events, such as outcome and onset date (REAC); and medical history (HIST). A relational database was constructed by integrating the four tables using the identification number as the primary key. Integration and management were performed using FileMaker Pro 18 Advanced (FileMaker, Santa Clara, CA), and an entity-relationship diagram was developed to illustrate the database structure. The DRUG dataset contains role codes designating each medication as a “suspected drug,” “concomitant drug,” or “interaction.” For this analysis, only entries identified as suspected drugs were retained.

Target drugs

This analysis focused on IVA, IVR, IVB, and IVF, which are approved in Japan. The use of bevacizumab for AMD treatment was reported in 146 cases (including three cases of cerebral infarctions) in the JADER database. Bevacizumab was excluded because it is used in nonapplicable uses. Pegaptanib was excluded because it was reported in only 36 cases (including three cases of cerebral infarction) and was discontinued in May 2020 [16].

Definition of adverse events

Adverse events were classified using the Medical Dictionary for Regulatory Activities (MedDRA, version 23.1) in accordance with the International Council for Harmonisation standards for the JADER database [17]. Reports specifying the intraocular route of administration were extracted for analysis. The conditions categorized under the ischemic cerebrovascular disease Standardized MedDRA Query (SMQ code: 20000063) were subsequently analyzed. The analysis included reports containing the following MedDRA preferred terms (PT): lacunar infarction (PT: 10051078), ischemic cerebral infarction (PT: 10060840), thrombotic cerebral infarction (PT: 10067347), embolic cerebral infarction (PT: 10060839), embolic stroke (PT: 10014498), cerebellar infarction (PT: 10008034), brain stem infarction (PT: 10006147), cerebral thrombosis (PT code: 10008132), and cerebral infarction (PT: 10008118). Disease progression, macular degeneration, and retinal vein occlusion were excluded because they were considered background patient information and included in the “HIST” table.

Statistical analysis

The number of reports and reporting odds ratios (RORs) were used for signal detection [18]. The ROR represents the ratio of the odds of reporting a specific adverse event for a particular drug to the odds of reporting all other adverse events for all other drugs in the database. It is calculated using a 2 × 2 contingency table, where “a” is the number of reports in which the patient received the drug of interest and experienced the adverse event, “b” is the number of reports where the patient received the drug of interest but did not experience the adverse event, “c” is the number of reports where the patient did not receive the drug of interest but experienced the adverse event, and “d” is the number of reports where the patient neither received the drug of interest nor experienced the adverse event. RORs were expressed as point estimates with 95% confidence intervals (CIs) (Table 1). The signal of a drug-adverse event combination was considered statistically significant when the estimated ROR and lower limit of the corresponding 95% CI were greater than 1. Positive signal identification required two or more cases.

**Table 1:** Two-by-two contingency table for calculation of reporting odds ratio (ROR) ROR = (a × d)/(b × c). 95% CI = e^[ln(ROR) ± 1.96√(1/a + 1/b + 1/c + 1/d)].

	Drug-induced disease	All other adverse events	Total
Targeted drug	a	b	a + b
All other drugs	c	d	c + d
Total	a + c	b + d	a + b + c + d

**Figure 1:**
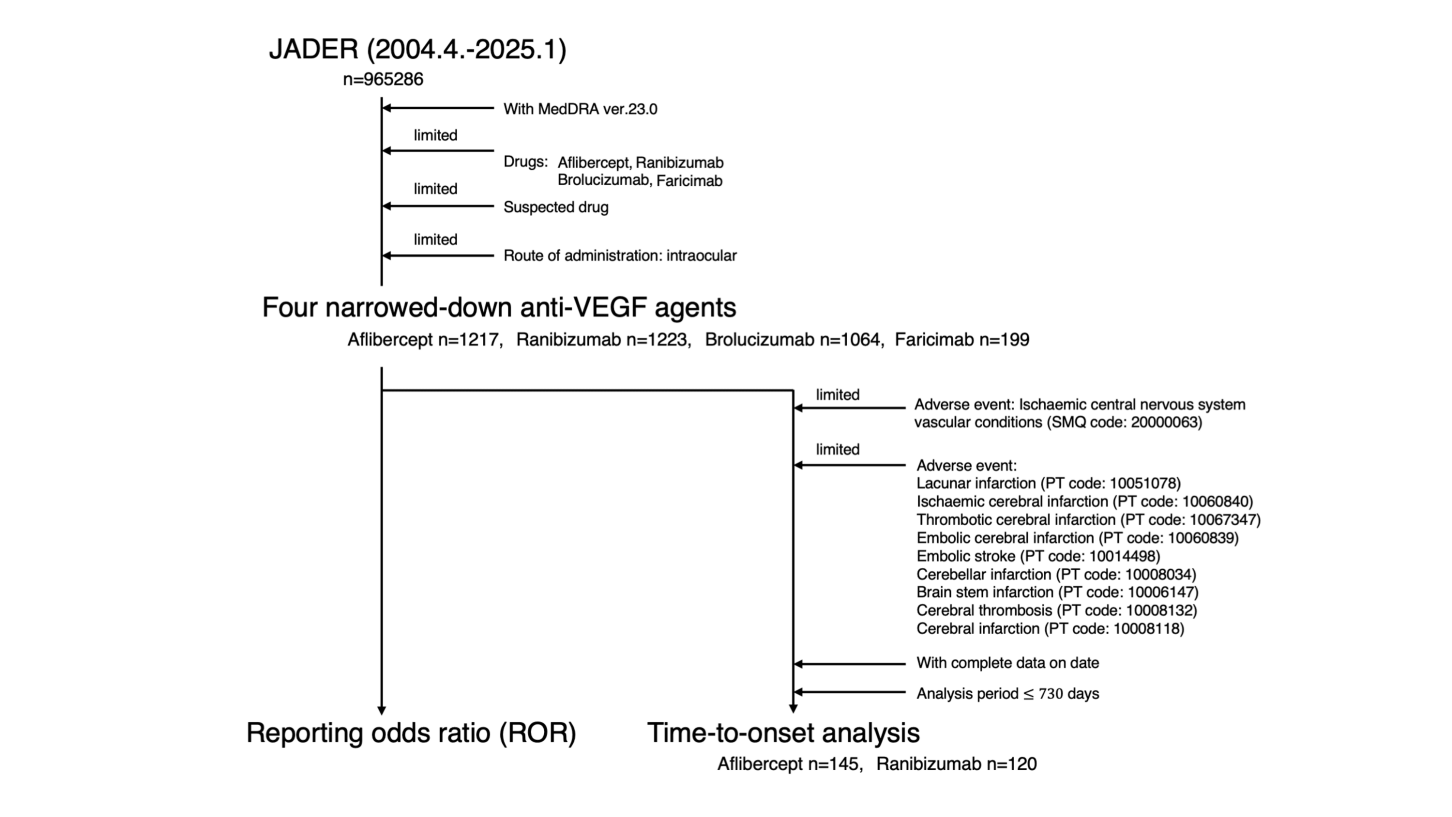
Flowchart depicting the process of data analysis VEGF: Vascular endothelial growth factor; JADER: Japanese Adverse Drug Event Report; MedDRA: Medical Dictionary for Regulatory Activities.

The adverse event-time cohorts were defined based on the following criteria: (1) the initiation date of IVA or IVR administration was available, (2) the date of onset of the adverse event was recorded, and (3) adverse reactions occurred after drug initiation. The median (interquartile range) interval between the date of treatment initiation and onset of adverse events was determined. Records in which the interval between treatment initiation and the onset of adverse drug reactions exceeded 730 days were considered to have low relevance to drug administration and were excluded from the analysis (Figure 1). Survival curves for anti-VEGF agents were compared using the Kaplan-Meier method, the log-rank test, and the generalized Wilcoxon test. Differences were considered significant at a p-value of <0.05. Statistical analyses were performed using JMP 11.2 (SAS Institute Inc., Cary, NC).

## Results

**Figure 2:**
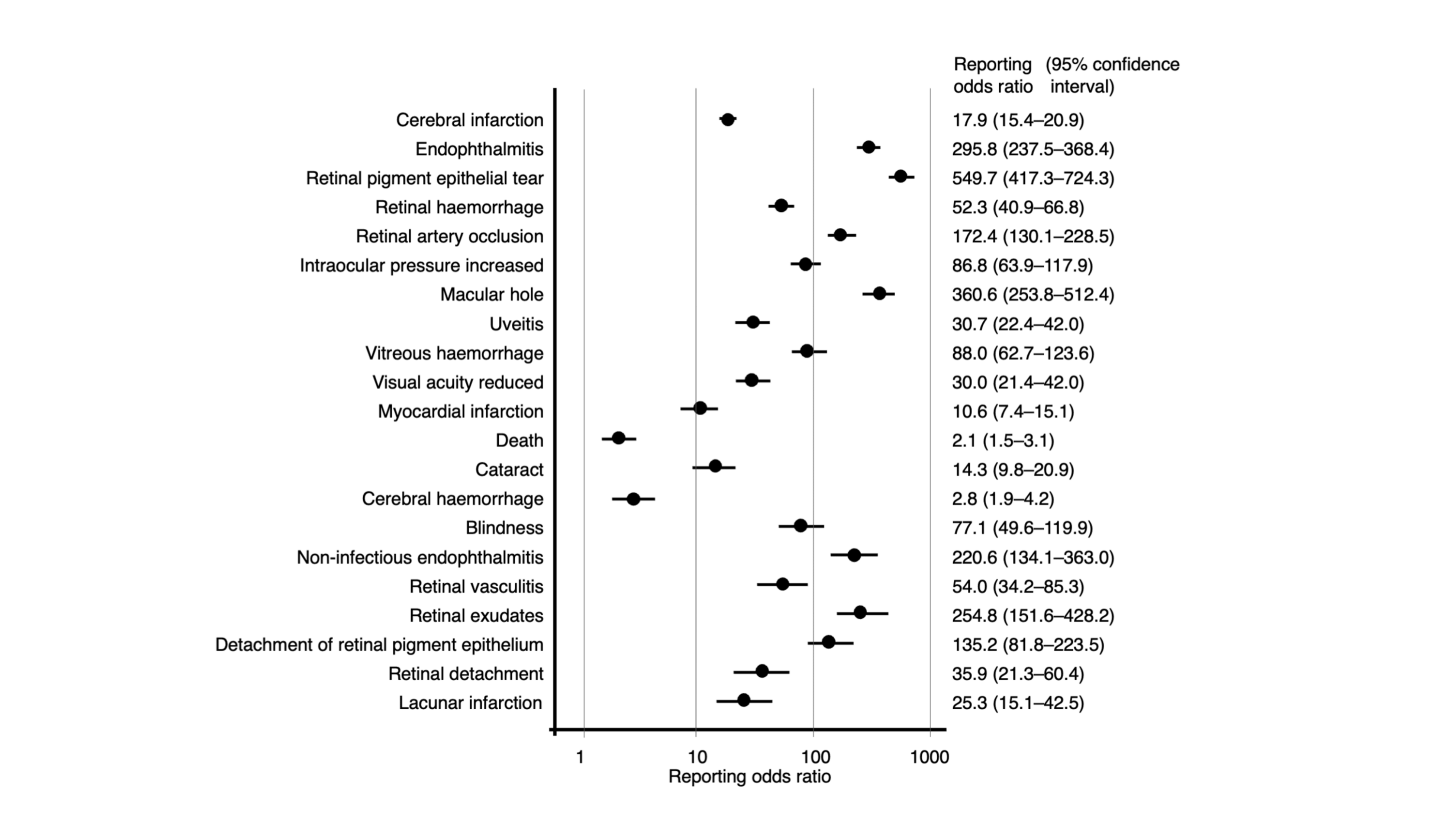
Reporting odds ratios for aflibercept

**Figure 3:**
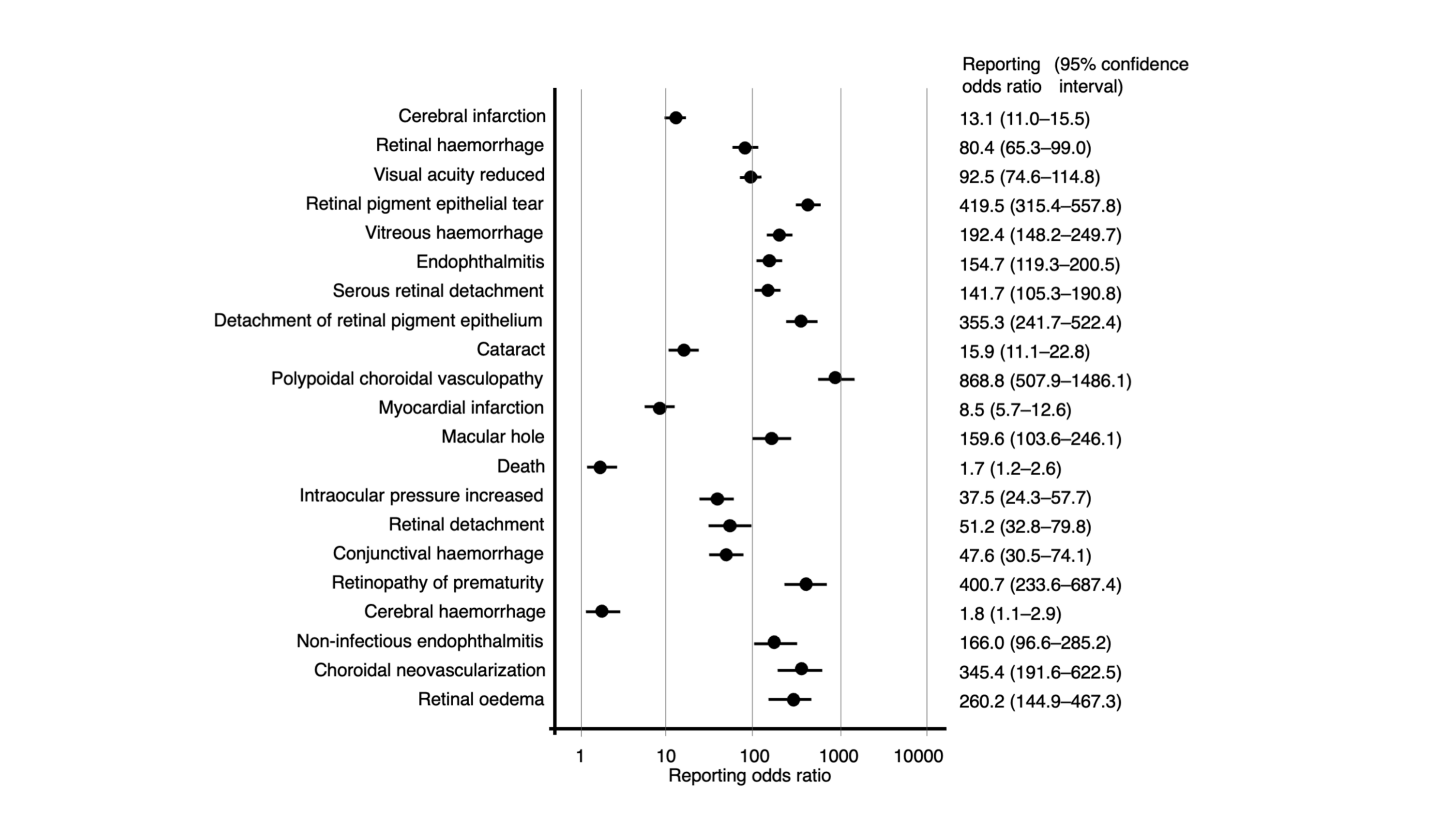
Reporting odds ratios for ranibizumab

**Figure 4:**
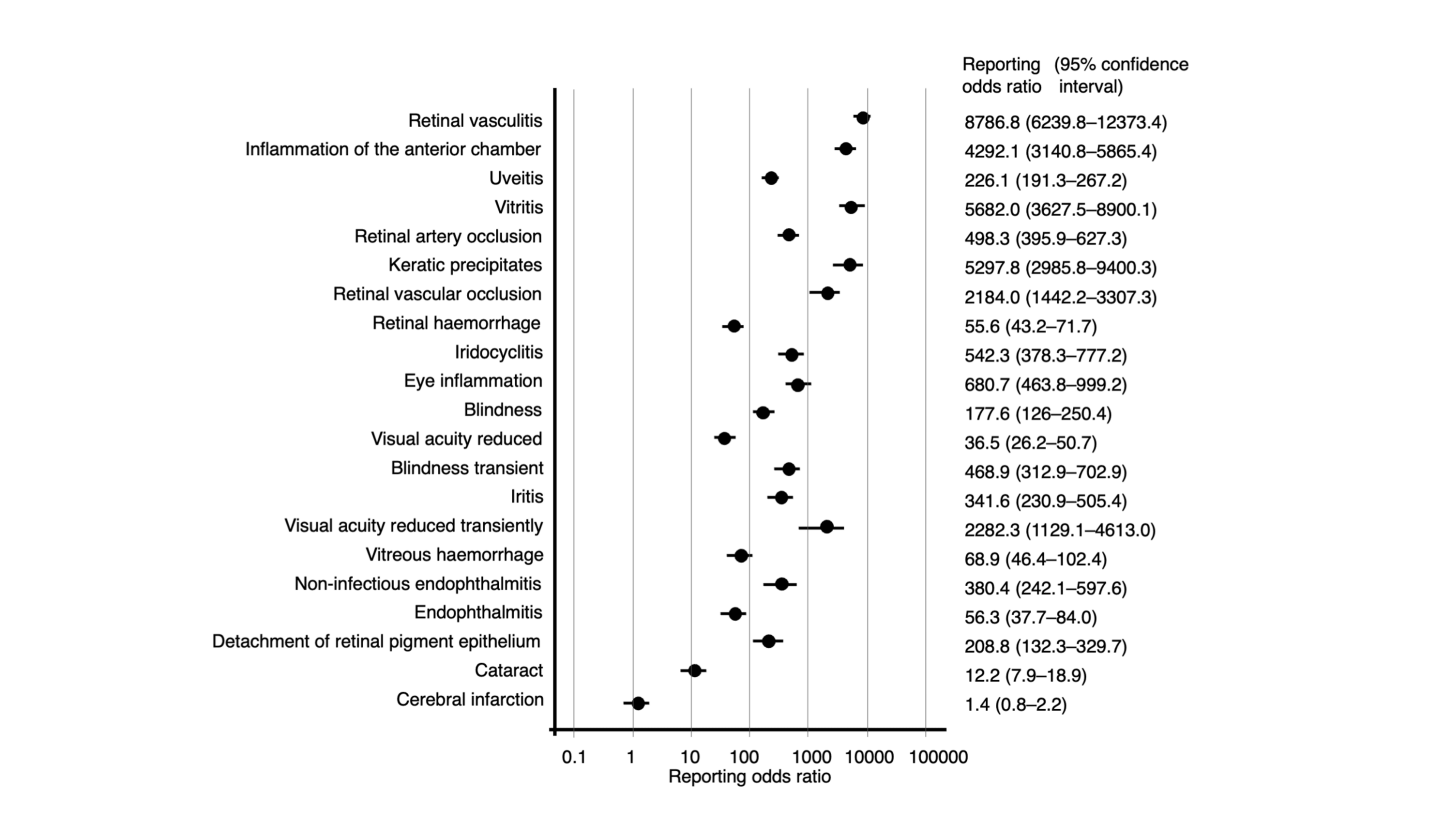
Reporting odds ratios for brolucizumab

**Figure 5:**
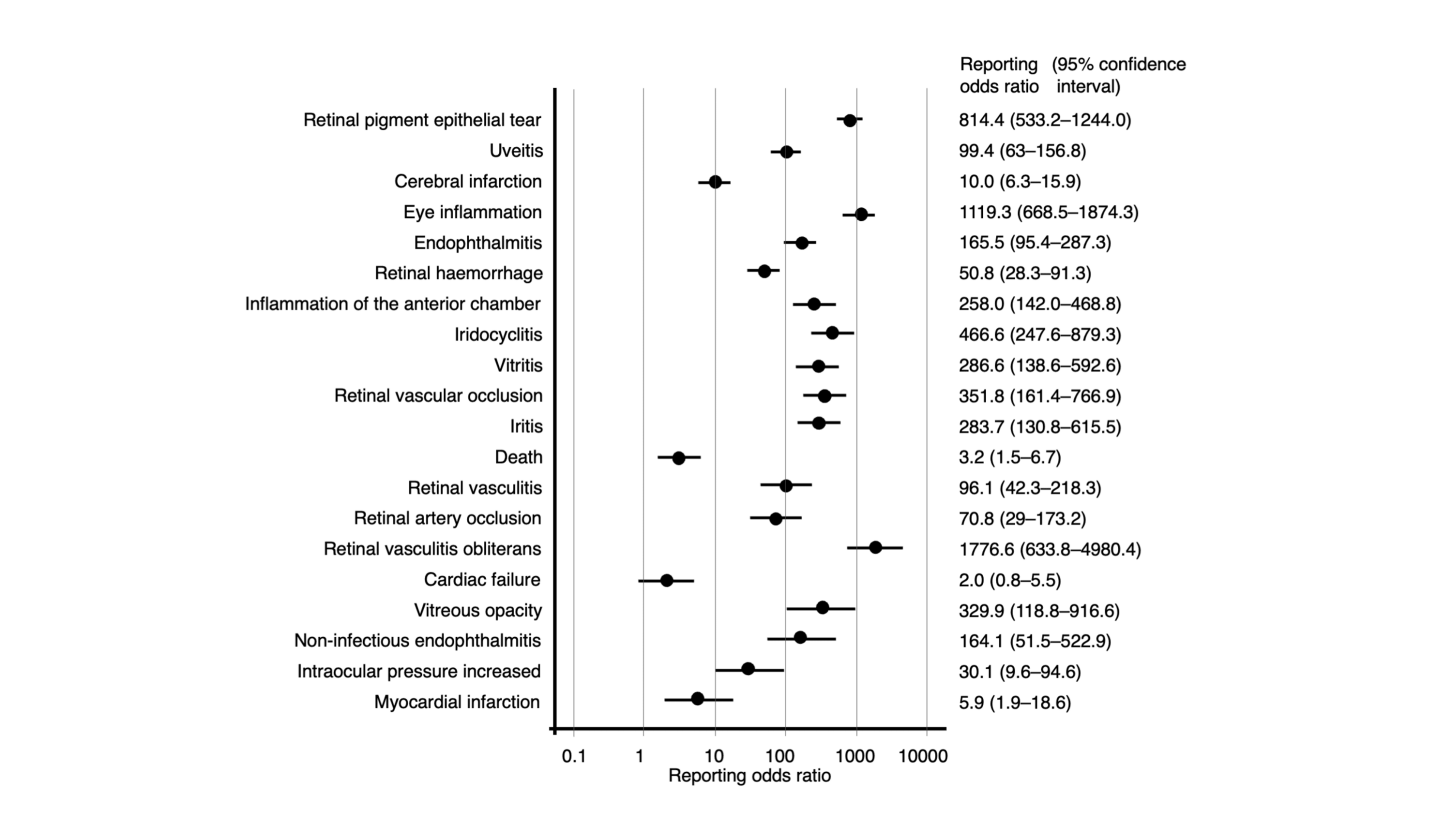
Reporting odds ratios for faricimab

The JADER database contains 965,286 reports submitted between April 2004 and January 2025. Among these, 1,217, 1,223, 1,064, and 199 adverse events were associated with IVA, IVR, IVB, and IVF, respectively. Overall, 43 records involved both IVA and IVR administration (none with cerebral infarction), 45 involved both IVA and IVB (two with cerebral infarction), 7 involved both IVA and IVF (none with cerebral infarction), 7 involved both IVR and IVB (none with cerebral infarction), 11 involved both IVA and IVF (none with cerebral infarction), and 1 involved both IVR and IVB (no cerebral infarction). There was no record in which both IVR and IVF were involved, and one record involved both IVB and IVF (no cerebral infarction). Both cases were included in the analyses. RORs were calculated for the top 20 reported adverse events summarized in Table 2 and Figures 2-5.

**Table 2:** Number of reports and reporting odds ratios for anti-VEGF agents *Case: Patients reporting adverse events related to anti-VEGF agents. ^†^Non-cases: Patients without adverse events related to anti-VEGF agents. VEGF: Vascular endothelial growth factor.

Drug name	Preferred terms	Preferred term code	Total (n)	Case*(n)	Non-case^†^(n)	Reporting odds ratio (ROR) (95% CI)
Aflibercept (IVA)						
Total			965286	1217	964069	
	Cerebral infarction	10018118	10662	200	10462	17.9 (15.4–20.9)
	Endophthalmitis	10014801	455	115	340	295.8 (237.5–368.4)
	Retinal pigment epithelial tear	10062971	222	87	135	549.7 (417.3–724.3)
	Retinal hemorrhage	10038867	1230	72	1158	52.3 (40.9–66.8)
	Retinal artery occlusion	10038827	356	61	295	172.4 (130.1–228.5)
	Intraocular pressure increased	10022806	493	47	446	86.8 (63.9–117.9)
	Macular hole	10051058	151	46	105	360.6 (253.8–512.4)
	Uveitis	10046851	1165	42	1123	30.7 (22.4–42.0)
	Vitreous hemorrhage	10047655	391	38	353	88.0 (62.7–123.6)
	Visual acuity reduced	10047531	1016	36	980	30.0 (21.4–42.0)
	Myocardial infarction	10028596	2485	32	2453	10.6 (7.4–15.1)
	Death	–	11002	29	10973	2.1 (1.5–3.1)
	Cataract	10007739	1608	28	1580	14.3 (9.8–20.9)
	Cerebral hemorrhage	10008111	7162	25	7137	2.8 (1.9–4.2)
	Blindness	10005169	252	22	230	77.1 (49.6–119.9)
	Noninfectious endophthalmitis	–	93	20	73	220.6 (134.1–363.0)
	Retinal vasculitis	10038905	318	20	298	54.0 (34.2–85.3)
	Retinal exudates	10038862	79	19	60	254.8 (151.6–428.2)
	Detachment of retinal pigment epithelium	10052501	125	18	107	135.2 (81.8–223.5)
	Retinal detachment	10038848	350	15	335	35.9 (21.3–60.4)
	Lacunar infarction	10051078	490	15	475	25.3 (15.1–42.5)
Ranibizumab (IVR)						
Total			965286	1223	964063	
	Cerebral infarction	10018118	10662	154	10508	13.1 (11.0–15.5)
	Retinal hemorrhage	10038867	1230	105	1125	80.4 (65.3–99.0)
	Visual acuity reduced	10047531	1016	99	917	92.5 (74.6–114.8)
	Retinal pigment epithelial tear	10062971	222	74	148	419.5 (315.4–557.8)
	Vitreous hemorrhage	10047655	391	73	318	192.4 (148.2–249.7)
	Endophthalmitis	10014801	455	71	384	154.7 (119.3–200.5)
	Serous retinal detachment	10040114	361	53	308	141.7 (105.3–190.8)
	Detachment of retinal pigment epithelium	10052501	125	38	87	355.3 (241.7–522.4)
	Cataract	10007739	1608	31	1577	15.9 (11.1–22.8)
	Polypoidal choroidal vasculopathy	–	54	28	26	868.8 (507.9–1486.1)
	Myocardial infarction	10028596	2485	26	2459	8.5 (5.7–12.6)
	Macular hole	10051058	151	25	126	159.6 (103.6–246.1)
	Death	–	11002	24	10978	1.7 (1.2–2.6)
	Intraocular pressure increased	10022806	493	22	471	37.5 (24.3−57.7)
	Retinal detachment	10038848	350	21	329	51.2 (32.8–79.8)
	Conjunctival hemorrhage	10010719	375	21	354	47.6 (30.5–74.1)
	Retinopathy of prematurity	10038933	60	20	40	400.7 (233.6–687.4)
	Cerebral hemorrhage	10008111	7162	16	7146	1.8 (1.1–2.9)
	Noninfectious endophthalmitis	–	93	16	77	166.0 (96.6–285.2)
	Choroidal neovascularization	–	53	16	37	345.4 (191.6–622.5)
	Retinal edema	10038886	61	15	46	260.2 (144.9–467.3)
Brolucizumab (IVB)						
Total			965286	1064	964222	
	Retinal vasculitis	10038905	318	279	39	8786.8 (6239.8–12373.4)
	Inflammation of the anterior chamber	–	252	200	52	4292.1 (3140.8–5865.4)
	Uveitis	10046851	1165	197	968	226.1 (191.3–267.2)
	Vitritis	10047663	150	127	23	5682.0 (3627.5–8900.1)
	Retinal artery occlusion	10038827	356	117	239	498.3 (395.9–627.3)
	Keratic precipitates	10053703	90	76	14	5297.8 (2985.8–9400.3)
	Retinal vascular occlusion	10038903	107	74	33	2184.0 (1442.2–3307.3)
	Retinal hemorrhage	10038867	1230	67	1163	55.6 (43.2–71.7)
	Iridocyclitis	10022941	132	48	84	542.3 (378.3–777.2)
	Eye inflammation	10015943	110	46	64	680.7 (463.8–999.2)
	Blindness	10005169	252	40	212	177.6 (126–250.4)
	Visual acuity reduced	10047531	1016	38	978	36.5 (26.2–50.7)
	Blindness transient	10005184	108	36	72	468.9 (312.9–702.9)
	Iritis	10022955	131	35	96	341.6 (230.9–505.4)
	Visual acuity reduced transiently	10047532	38	27	11	2282.3 (1129.1–4613.0)
	Vitreous hemorrhage	10047655	391	27	364	68.9 (46.4–102.4)
	Noninfectious endophthalmitis	–	93	27	66	380.4 (242.1–597.6)
	Endophthalmitis	10014801	455	26	429	56.3 (37.7–84.0)
	Detachment of retinal pigment epithelium	10052501	125	23	102	208.8 (132.3–329.7)
	Cataract	10007739	1608	21	1587	12.2 (7.9–18.9)
	Cerebral infarction	10018118	10662	16	10646	1.4 (0.8–2.2)
Faricimab (IVF)						
Total			965286	199	965087	
	Retinal pigment epithelial tear	10062971	222	28	194	814.4 (533.2–1244.0)
	Uveitis	10046851	1165	21	1144	99.4 (63.0–156.8)
	Cerebral infarction	10018118	10662	20	10642	10.0 (6.3–15.9)
	Eye inflammation	10015943	110	19	91	1119.3 (668.5–1874.3)
	Endophthalmitis	10014801	455	14	441	165.5 (95.4–287.3)
	Retinal hemorrhage	10038867	1230	12	1218	50.8 (28.3–91.3)
	Inflammation of the anterior chamber	–	252	12	240	258.0 (142.0–468.8)
	Iridocyclitis	10022941	132	11	121	466.6 (247.6–879.3)
	Vitritis	10047663	149	8	141	286.6 (138.6–592.6)
	Retinal vascular occlusion	10038903	107	7	100	351.8 (161.4–766.9)
	Iritis	10022955	131	7	124	283.7 (130.8–615.5)
	Death	–	11002	7	10995	3.2 (1.5–6.7)
	Retinal vasculitis	10038905	318	6	312	96.1 (42.3–218.3)
	Retinal artery occlusion	10038827	356	5	351	70.8 (29–173.2)
	Retinal vasculitis obliterans	–	19	5	14	1776.6 (633.8–4980.4)
	Cardiac failure	10007554	9578	4	9574	2.0 (0.8–5.5)
	Vitreous opacity	–	64	4	60	329.9 (118.8–916.6)
	Noninfectious endophthalmitis	–	93	3	90	164.1 (51.5–522.9)
	Intraocular pressure increased	10022806	493	3	490	30.1 (9.6–94.6)
	Myocardial infarction	10028596	2485	3	2482	5.9 (1.9–18.6)

The RORs for the top five adverse events associated with IVA were as follows: cerebral infarction (PT code: 10008118), 17.9 (95% CI, 15.4-20.9); endophthalmitis (PT code: 10014801), 295.8 (95% CI, 237.5-368.4); retinal pigment epithelial tear (PT code: 10062971), 549.7 (95% CI, 417.3-724.3); retinal hemorrhage (PT code: 10038867), 52.3 (95% CI, 40.9-66.8); and retinal artery occlusion (PT code: 10038827), 172.4 (95% CI, 130.1-228.5). For IVR, the top five adverse events and their corresponding RORs were as follows: cerebral infarction, 13.1 (95% CI, 11.0-15.5); retinal hemorrhage, 80.4 (95% CI, 65.3-99.0); visual acuity reduced (PT code: 10047531), 92.5 (95% CI, 74.6-114.8); retinal pigment epithelial tear, 419.5 (95% CI, 315.4-557.8); and vitreous hemorrhage (PT code: 10047655), 192.4 (95% CI, 148.2-249.7). The top five adverse events of IVB and their RORs were as follows: retinal vasculitis (PT code: 10038905), 8786.8 (95% CI, 6239.8-12373.4); inflammation of the anterior chamber, 4292.1 (95% CI, 3140.8-5865.4); uveitis (PT code: 10046851), 226.1 (95% CI, 191.3-267.2); vitritis (PT code: 10047663), 5682.0 (95% CI, 3627.5-8900.1); and retinal artery occlusion, 498.3 (95% CI, 395.9-627.3). For IVF, the top five adverse events and their corresponding RORs were as follows: retinal pigment epithelial tear, 814.4 (95% CI, 533.2-1244.0); uveitis, 99.4 (95% CI, 63.0-156.8); cerebral infarction, 10.0 (95% CI, 6.3-15.9); eye inflammation (PT code: 10015943), 1119.3 (95% CI, 668.5-1874.3); and endophthalmitis, 165.5 (95% CI, 95.4-287.3).

**Figure 6:**
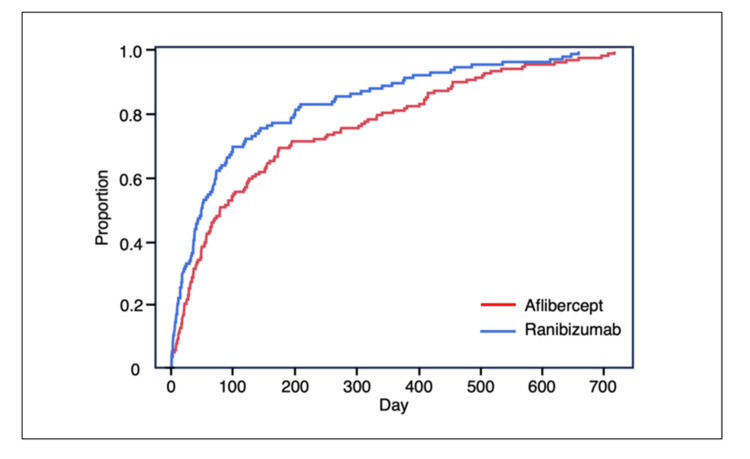
Kaplan-Meier plot of cerebral infarction for aflibercept and ranibizumab Statistical analysis was performed using the log-rank test (p = 0.0100) and the generalized Wilcoxon test (p = 0.0067).

The numbers of reported cerebral infarctions associated with IVB and IVF were 16 and 20, respectively. The ROR for cerebral infarction associated with IVB, which was the 22nd most frequently reported adverse event overall, was 1.4 (95% CI, 0.8-2.2). The proportions of cerebral infarction among the adverse events associated with IVA, IVR, and IVB were 16.4%, 12.6%, and 1.5%, respectively. In the adverse event-time analysis, 145 and 120 reports were analyzed for the IVA and IVR groups, respectively. The median (interquartile range) onset times of cerebral infarction were 79.0 (28.0-274.0) days for IVA and 48.5 (14.0-142.5) days for IVR. The survival time analysis using the log-rank and generalized Wilcoxon tests revealed a significant difference in the survival distributions(log-rank test: p = 0.0100, generalized Wilcoxon test: p = 0.0067). IVB and IVF were associated with 12 and 11 cases of cerebral infarction, respectively; the intergroup difference was not significant (Figure 6).

## Discussion

This study evaluated the association between anti-VEGF agents and the occurrence of adverse events using the JADER database. In Japan, intraocular injection therapy with anti-VEGF agents is widely used to treat macular degeneration and macular edema. Although cerebral infarction is a rare adverse event associated with these drugs, it is a serious and potentially fatal complication, particularly in elderly patients with AMD. Therefore, understanding the safety profiles of these agents is essential for clinical decision-making.

The accompanying product information for IVA, IVR, IVB, and IVF includes precautions for patients with risk factors for stroke, such as a history of cerebrovascular events (cerebral infarction and cerebral hemorrhage) or transient ischemic attack [19-22]. Our analysis detected significant ROR signals for cerebral infarction among the top 20 most frequently reported adverse events associated with anti-VEGF agents. The presence of statistically significant ROR signals for all four drugs indicates an association between these agents and adverse events, highlighting the importance of early intervention and careful patient monitoring in clinical practice.

VEGF inhibitors exert anticoagulant effects through increased levels of plasminogen activator inhibitor-1 (PAI-1), a thrombus-promoting mediator, and enhanced expression of von Willebrand factor in cultured cells. In addition, antiplatelet effects mediated by NO and PGI2 and increased expression of urokinase, tissue plasminogen activator, and urokinase receptor, which are primarily expressed in endothelial cells, contribute to the anticoagulant effects [23-26]. However, in vivo, the antithrombotic effects of VEGF may predominate [27,28]. These inhibitions can lead to thrombosis [4].

A potential mechanism linking VEGF inhibitors to cerebral infarction is the inhibition of VEGF receptors (VEGFR) signaling, leading to increased vasoconstriction through reduced nitric oxide and prostacyclin production, which can cause hypertension [4,29,30]. Additionally, VEGF plays a crucial role in repairing damaged endothelial surfaces; its inhibition may expose the subendothelial tissue and induce endothelial cell apoptosis, triggering the coagulation cascade and thrombus formation [31].

Among the agents evaluated, IVA, IVR, and IVB were associated with over 1,000 reported cases, and the corresponding proportions of cerebral infarction among the adverse events associated with these drugs were 16.4%, 12.6%, and 1.5%. Although the related literature is scarce, these findings may serve as a benchmark for future investigations. IVA and IVR were introduced in 2012 and 2009, respectively, while IVB and IVF were launched more recently (2020 and 2022, respectively) [19-22]. As more data accumulate, a more precise risk evaluation for IVB and IVF will become possible.

Notably, this study suggests that IVR may be associated with an earlier onset of cerebral infarction than IVA. This observation may be explained by structural differences. IVA contains an Fc region, which prolongs its systemic half-life by avoiding lysosomal degradation and renal clearance, whereas IVR does not have this region, resulting in shorter systemic exposure [8]. Consequently, IVR may be associated with a shorter duration of adverse effects; however, this requires further investigation. Based on these findings, implementing early patient evaluation and monitoring systems, such as neurological screening, coagulation assessment at treatment initiation, and rapid-response protocols for neurological symptoms, may improve patient safety during IVR treatment.

It was necessary to consider right truncation when evaluating the time to onset of adverse events. We determined an analysis period of 730 days after the start of administration to focus on the onset of adverse events within two years of the patients’ first prescription. To the best of our knowledge, there are currently no standardized criteria for defining the observation period in time-to-onset analyses. The appropriate duration can vary substantially, ranging from several weeks to several years, depending on the drug in question and the specific adverse event under investigation [32,33]. In the present study, because the timing of infarction associated with anti-VEGF agents is not clearly understood, we selected a two-year observation period. As the duration of the observation period increases, the potential impact of non-drug-related factors on the patient’s clinical status becomes increasingly difficult to exclude. Hence, caution is warranted when extending the time-to-onset analysis period using spontaneous reporting data, and analyses beyond two years were not conducted in this study. Anti-VEGF agents are typically administered once a month for approximately three months, and their systemic half-life is approximately 4.4-11.3 days [19-22]. Considering this, we also evaluated the time to onset of adverse events within 180 days (six months) after the initiation of treatment (data not shown). The median time to onset (interquartile range) was 47 days (20-92 days) for IVA and 36 days (10-69 days) for IVR (log-rank test: p = 0.00867; generalized Wilcoxon test: p = 0.0286), findings that align with the results of the two-year analysis.

IVA and IVR were introduced in 2012 and 2009, respectively. As shown in Figure 1 and Table 2, the number of cases used for the calculation of ROR was 1,217 for IVA and 1,223 for IVR, while the number of cases used for the time-to-onset analysis was 145 and 120, respectively. Although there is a three-year difference in the marketing durations of IVA and IVR, the number of reports does not differ substantially, suggesting that this discrepancy is unlikely to have affected the present comparison. Furthermore, the Weber effect, which describes the tendency for spontaneous adverse event reports to increase shortly after a drug’s approval, plateau after approximately two years, and subsequently decline [34], may have minimal impact in this analysis, as both drugs have been on the market for more than 10 years.

Specific patient characteristics may contribute to the risk of cerebral infarction. Nevertheless, it is well recognized that information on underlying conditions and other patient background factors recorded in the JADER database is often incomplete. In the current dataset, 102 of the 246 patients treated with IVA had hypertension, and 31 had hyperlipidemia, while among the 189 patients treated with IVR, 92 had hypertension, and 16 had hyperlipidemia. Although these comorbidities could be associated with the onset of cerebral infarction, their precise impact warrants clarification through more rigorously controlled epidemiological investigations.

This study had several limitations. The JADER database is a spontaneous reporting system and is inherently unsuitable for precise risk quantification because of issues such as under-reporting, over-reporting, missing data, lack of detailed patient characteristics, absence of comparator groups, influence of confounding factors, and reporting bias. Spontaneous reporting systems provide only a rough indication of signal strength rather than an absolute measure of risk; therefore, these findings should be cautiously interpreted. Further confirmatory studies, including prospective observational research and mechanistic investigations, are required to validate these results. Despite these limitations, this study provides practical insights, particularly considering the lack of prior analyses focusing on the timing of cerebral infarction after anti-VEGF therapy. Our findings may contribute to improved drug safety assessments and support the development of personalized treatment strategies in ophthalmology.

This study used the JADER database to explore the association between anti-VEGF agents and the risk of cerebral infarction. However, the JADER database, as a spontaneous reporting system, has inherent limitations, such as under-reporting, over-reporting, missing data, lack of detailed patient background information, absence of control groups, and the influence of confounding factors and bias. Therefore, these findings should be interpreted with caution because spontaneous reporting systems provide only signal strength rather than an absolute measure of risk. Further confirmatory studies, including large-scale observational or basic research, are warranted to validate these results. Despite these limitations, our analysis offers practical insights, particularly given the paucity of studies on the timing of cerebral infarction onset associated with anti-VEGF therapy and may contribute to a better understanding of drug safety in clinical practice.

## Conclusions

Intraocular injection therapy with anti-VEGF agents for macular degeneration and macular edema is widely used in Japan. Although cerebral infarction is a rare adverse effect of these drugs, it can result in mortality, particularly in elderly patients with macular degeneration. This study suggests a notable difference in the timing of cerebral infarction associated with IVA and with IVR. Cerebral infarction may develop earlier with IVR use than with IVA use. Therefore, a more cautious management strategy is warranted when using IVR. These findings may contribute to improved risk assessment during drug selection and emphasize the importance of personalized medicine in clinical practice.
